# Microcyclophotocoagulation in Glaucoma Treatment: A Medium-Term Follow-Up Study

**DOI:** 10.3390/jcm12134342

**Published:** 2023-06-28

**Authors:** Bartłomiej Bolek, Adam Wylęgała, Edward Wylęgała

**Affiliations:** 1 Chair and Clinical Department of Ophthalmology, School of Medicine in Zabrze, Medical University of Silesia in Katowice, District Railway Hospital, 40-760 Katowice, Poland; 2 Health Promotion and Obesity Management, Pathophysiology Department, Medical University of Silesia in Katowice, 40-752 Katowice, Poland

**Keywords:** cyclodestruction, glaucoma, microcyclophotocoagulation, transscleral cyclophotocoagulation, micropulse

## Abstract

Background: This study aimed to assess the effectiveness and safety of transscleral microcyclophotocoagulation (µCPC) in patients with glaucoma for eighteen consecutive months. Methods: Sixty-one patients (64 eyes) with primary and secondary glaucoma were enrolled to undergo µCPC (diode laser FOX 810, A.R.C. Laser, Nuremberg, Germany). The primary outcome measures were intraocular pressure (IOP) reduction, success rates, glaucoma medication use, and visual acuity after µCPC. An IOP reduction of 20% compared to the baseline value without re-intervention was considered a successful treatment. Complete success was defined as cessation of antiglaucoma medications. Secondary outcome measures included intraoperative and postoperative complications. Measurements were performed preoperatively and at the first week, and 1, 3, 6, 12, and 18 months postoperatively. Results: The mean ± SD values of IOP preoperatively at 1 day, 1 week, 1, 3, 6, 12, and 18 months postoperatively were 25.1 ± 8.4 mmHg, 17.3 ± 4.5 mmHg (*p* < 0.001), 16.5 ± 6.1 mmHg (*p* < 0.001), 20.5 ± 8.3 mmHg (*p* < 0.001), 17.1 ± 6.2 mmHg (*p* < 0.001), 18.0 ± 7.1 mmHg (*p* < 0.001), 15.8 ± 3.2 mmHg (*p* < 0.001), and 17.0 ± 5.9 mmHg (*p* < 0.001), respectively. The mean IOP at the last follow-up was reduced by 32.5%. The decrease in the number of antiglaucoma medications was statistically significant at each follow-up visit compared to the baseline. The qualified success rate was 38.5%. Two patients at 18-month follow-up did not require the use of antiglaucoma medications—complete success rate—3.1%. During the follow-up period, twenty-five eyes (39.1%) that required retreatment due to nonachievement of the target IOP were considered as failures. Eleven patients (12 eyes—18.8%) were lost to follow-up. A total of 26 patients (27 eyes) were evaluated 18 months after µCPC. Hypotony was observed in one patient (1.6%) and uveitis in two patients (3.1%) after the procedure. There were no other significant intraoperative or postoperative complications observed. Conclusions: The µCPC is well tolerated and safe for reducing IOP in glaucoma patients in medium-term follow-up; however, success is moderate. Randomized, larger studies are needed to confirm the obtained results.

## 1. Introduction

Cyclodestructive methods are used to treat moderate and severe forms of glaucoma [1,2,3]. These methods reduce intraocular pressure (IOP) by decreasing aqueous humor production by partially damaging the non-pigmented epithelium of the ciliary body. Microcyclophotocoagulation (µCPC) for glaucoma treatment, compared to the commonly used diode laser cyclodestruction, can limit heat accumulation and avoid coagulation temperatures, resulting in less disruption of the non-pigmented epithelium and adjacent tissues due to laser pulse segmentation [2,4,5,6,7]. This results in an improvement in the safety of this type of treatment. Until now, only one study has been published, in a non-English journal, where µCPC was performed on African patients with a diode laser FOX 810 (A.R.C. Laser, Nuremberg, Germany) with a success rate of around 65% at 9 months follow-up [8]. All other literature about micropulse cyclophotocoagulation published so far refers to the use of different devices. 

In this medium-term prospective single-center study, the largest so far, the objective is to verify the effectiveness and safety of the treatment in glaucoma patients who underwent the µCPC procedure using diode laser FOX in 18 consecutive months of follow-up. The primary outcome measures included the IOP reduction, success rates, glaucoma medication use, and visual acuity following µCPC. Secondary outcome measures were any complications occurring during the procedure or in the postoperative phase.

## 2. Materials and Methods

This was a non-randomized, prospective, single-arm, single-center follow-up clinical study. It received approval from the institutional review board of Medical University of Silesia (KNW/0022/KB1/78/18). Patients were recruited between February 2021 and November 2021. All enrolled patients provided written informed consent prior to participating in the study.

The inclusion criteria for the study were: adult patients (≥18 years), uncontrolled glaucoma (IOP > 21 mmHg, despite maximum tolerated doses of antiglaucoma medications), or intolerance to glaucoma medication despite well-controlled IOP. Exclusion criteria included pregnant women and patients below 18 years of age. Comprehensive ophthalmic examinations were conducted, which involved measurements of IOP using the standard Goldmann applanation tonometer (GAT), assessment of the number of antiglaucoma medications, and determination of best-corrected logMAR visual acuity. These examinations were performed preoperatively and at 1 day, 1 week, 1, 3, 6, 12, and 18 months postoperatively. The IOP measurements adhered to the guidelines set forth by the World Glaucoma Association for the design and reporting of glaucoma surgical trials [9]. This study considered a treatment successful (qualified success) if there was a 20% reduction in IOP, and IOP remained below 21 mmHg during the 18-month follow-up visits compared to the baseline value without requiring additional surgical intervention. Complete success was defined as cessation of antiglaucoma medications. Failure was defined as IOP not being reduced by at least 20% from the baseline and above 21 mmHg (in two consecutive visits), and/or the necessity for further glaucoma surgical intervention. In cases where IOP was not sufficiently lowered during follow-up and the number of antiglaucoma medications was reduced compared to the baseline, additional medications were added. If needed, further glaucoma surgery was performed based on the patient’s clinical conditions. This study group underwent the following procedures as re-interventions: transscleral cyclophotocoagulation (TSCPC), transscleral microcyclophotocoagulation (µCPC), ultrasound ciliary plasty (UCP), trabeculectomy, and Ahmed valve implantation. 

µCPC was performed using diode laser FOX 810 (A.R.C. Laser, Nuremberg, Germany) in the operating room under peribulbar anesthesia. In accordance with the manufacturer’s recommendations, the laser parameters used were a power of 2 W, 31.3% duty cycle (“on” period of 500 µs/“off” period of 1.0 ms) and 90–120 s per hemisphere. The procedure involved continuous sliding of a laser probe along the limbus, 1–1.5 mm posterior, avoiding the 3 and 9 o’clock meridians, areas of scleral thinning, and previous glaucoma surgeries. All procedures were performed by the same surgeon (B.B.). Based on the postoperative intraocular pressure (IOP) readings, the preoperative antiglaucoma medication regimen was either maintained or adjusted. Following the surgery, patients received topical treatment with ofloxacin (five times a day for two weeks) and dexamethasone (five times a day for two weeks, followed by three times a day for two weeks).

Statistical analyses were conducted using Statistica Software version 13 (TIBCO Software Inc., Palo Alto, CA, USA). The comparison of dataset groups for a specific parameter was performed using either the Wilcoxon signed-rank test or paired *t*-test, depending on the distribution of the data. Kaplan–Meier survival curves were utilized to assess the qualified and complete success of the treatment over time. The results of IOP are visually presented through a scatter plot, depicting preoperative IOP on the x-axis versus 18-month postoperative IOP on the y-axis. A *p*-value of ≤0.05 was considered statistically significant.

## 3. Results

Sixty-one patients (64 eyes) diagnosed with primary and secondary glaucoma were included in the study for µCPC. Detailed patient characteristics can be found in Table 1. The mean energy delivered was 135.9 ± 27.0 J. During the follow-up period, twenty-five eyes (39.1%) were re-treated due to nonachievement of the target IOP and were considered failures. In eighteen eyes (26.6%), TSCPC surgery was performed on average 9 months after µCPC. Three patients (4.7%) were re-treated with UCP on average 8 months after µCPC. Two patients (3.1%) needed trabeculectomy 3 months after µCPC. Two patients (3.1%) needed Ahmed valve implantation on average 6 months after µCPC. Eleven patients (12 eyes—18.8%) were lost to follow-up during the study period. Among them, five patients died for reasons unrelated to glaucoma, while six patients discontinued their participation in the study visits due to reasons of a medical or non-medical nature that were unrelated to glaucoma. Consequently, a total of 26 patients (corresponding to 27 eyes) underwent evaluation at the 18-month follow-up following µCPC.

**Figure 1:**
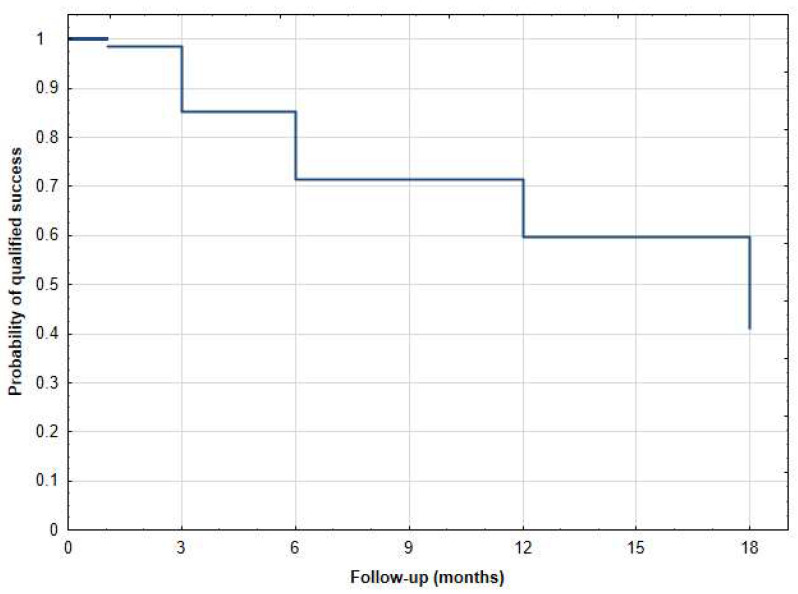
Kaplan–Meier survival curve of qualified success after µCPC—18-month follow-up.

**Figure 2:**
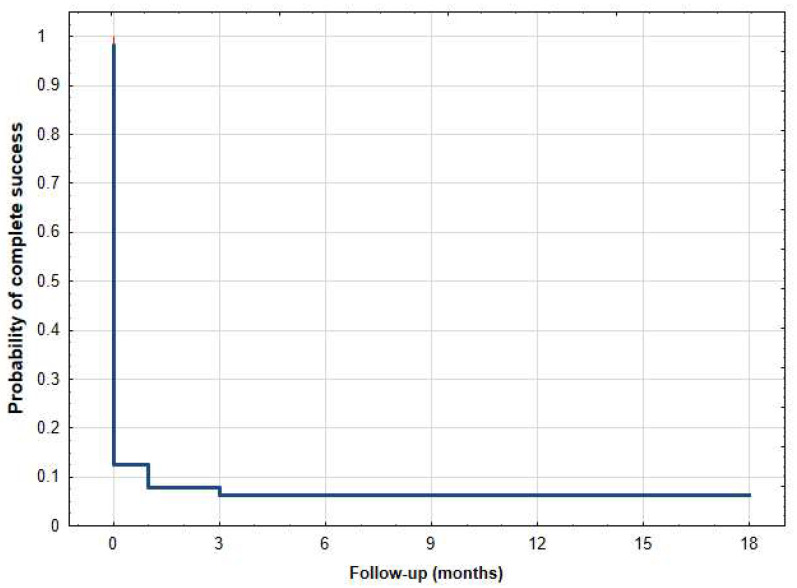
Kaplan–Meier survival curve of complete success after µCPC—18-month follow-up.

**Figure 3:**
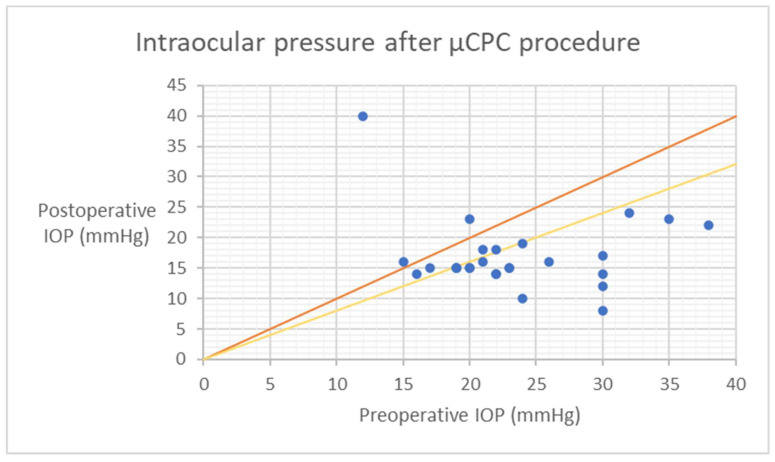
Scatter plot as preoperative IOP (x-axis) versus 18 months postoperative IOP (y-axis). The orange diagonal line at a 45-degree angle (y = x) illustrates patients with a reduction in IOP after surgery (right lower half) compared to those with an increase in IOP (left upper half) or no change. Yellow slope diagonal line represents 20% IOP reduction. µCPC—transscleral microcyclophotocoagulation, IOP—intraocular pressure.

The mean ± SD values of IOP preoperatively at 1 day, 1 week, 1, 3, 6, 12, and 18 months postoperatively were 25.1 ± 8.4 mmHg, 17.3 ± 4.5 mmHg (*p* < 0.001), 16.5 ± 6.1 mmHg (*p* < 0.001), 20.5 ± 8.3 mmHg (*p* < 0.001), 17.1 ± 6.2 mmHg (*p* < 0.001), 18.0 ± 7.1 mmHg (*p* < 0.001), 15.8 ± 3.2 mmHg (*p* < 0.001), and 17.0 ± 5.9 mmHg (*p* < 0.001), respectively (Table 2). The mean IOP at the last follow-up was reduced by 32.5% (Table 2). The qualified success rate was 38.5% (20/52 eyes). Among the subset of patients who did not achieve qualified success, a total of twenty-five eyes necessitated additional surgical interventions. Additionally, seven patients did not attain a minimum 20% reduction in intraocular pressure (IOP) from baseline. At the 18-month follow-up, two patients no longer required the use of antiglaucoma medications, resulting in a complete success rate of 3.1%. The Kaplan–Meier survival curves (Figure 1 and Figure 2) and the scatter plot (Figure 3) provide visual representations of the data. It is important to note that patients who were lost to follow-up were excluded from the analysis of success rates.

The mean ± SD values of the number of antiglaucoma medications preoperatively and at 1 day, 1 week, 1, 3, 6, 12 and 18 months postoperatively were 4.2 ± 1.0, 2.4 ± 1.1 (*p* < 0.001), 2.6 ± 1.1 (*p* < 0.001), 2.7 ± 1.1 (*p* < 0.001), 3.0 ± 1.2 (*p* < 0.001), 3.0 ± 1.1 (*p* < 0.001), 3.3 ± 1.0 (*p* < 0.001), and 3.3 ± 1.1 (*p* < 0.001), respectively (Table 2). Before undergoing µCPC, a total of twenty-seven eyes necessitated systemic carbonic anhydrase administration. At the 18-month follow-up, eight eyes maintained the same number of antiglaucoma medications as at baseline. Conversely, the remaining patients exhibited a reduction in the number of antiglaucoma medications at the last follow-up visit compared to baseline. Notably, among these nineteen cases, fifteen eyes had previously required systemic carbonic anhydrase treatment. None of the patients exhibited an increase in the number of antiglaucoma medications compared to the preoperative regimen during the last visit. In some instances, systemic carbonic anhydrase was added during follow-up immediately before surgical re-intervention to lower intraocular pressure. These patients were considered as treatment failures. None of the eyes necessitated systemic carbonic anhydrase at the 18-month follow-up visit.

The best-corrected logMAR visual acuities ± SD values preoperatively, at 1 week, 1, 3, 6, 12, and 18 months postoperatively were 1.01 ± 0.93, 1.05 ± 0.94 (*p* = 0.149), 0.99 ± 0.92 (*p* = 0.931), 1.03 ± 0.92 (*p* = 0.876), 1.13 ± 0.98 (*p* = 0.258), 1.11 ± 0.96 (*p* = 0.140), and 1.26 ± 0.97 (*p* = 0.363), respectively. A statistically significant reduction was observed in both IOP and the number of antiglaucoma medications when compared to the baseline (Table 2). The best-corrected logMAR visual acuity remained statistically unchanged. 

**Figure 4:**
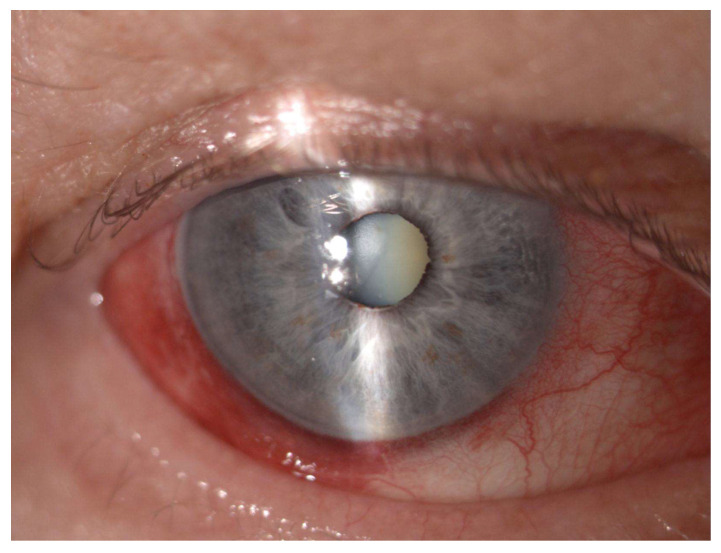
Postoperative subconjunctival hemorrhage after µCPC.

Table 3 lists the intraoperative and postoperative complications encountered during the study. Hypotony was observed in one patient (1.6%) and uveitis occurred in two patients (3.1%) after the procedure. Intraoperatively subconjunctival hemorrhage occurred in thirty-eight of our patients (59.4%), while postoperative conjunctival hyperemia in forty-four patients (68.7%) (Figure 4). There were no other significant intraoperative or postoperative complications observed.

## 4. Discussion

To summarize, this is the largest medium-term prospective single-center study of glaucoma patients who underwent µCPC procedures using diode laser FOX 810. At the final follow-up, the mean intraocular pressure (IOP) demonstrated a reduction exceeding more than a quarter. The qualified success rate was observed to be moderate. The occurrence of side effects was consistent with previously published findings [10,11,12,13,14,15]. 

Reducing intraocular pressure (IOP) remains the primary and evidence-based approach for the management of glaucoma [16,17]. This can be accomplished through various strategies, including the reduction of aqueous humor production and enhancement of its outflow using pharmacological and surgical interventions, alone or in combination. A commonly employed method for reducing aqueous humor production involves targeted damage to the non-pigmented epithelium of the ciliary body using techniques such as laser photocoagulation, cryotherapy, or ultrasound energy. Among these procedures, transscleral cyclophotocoagulation (TSCPC) is recognized as the most prevalent and efficacious method. The mechanism of action of TSCPC is the destruction of the pigmented ciliary body epithelium with indirect destruction of the non-pigmented cell and an increase in the uveoscleral outflow [18,19]. This method is mainly used in severe forms of refractory glaucoma, when previous pharmacological or surgical treatments (filtration or seton) were not successful [20]. Cyclodestruction techniques, including transscleral cyclophotocoagulation (TSCPC), are associated with two primary drawbacks in glaucoma treatment. Firstly, there is limited selectivity of the target tissue, leading to potential damage to adjacent structures. Secondly, predicting the therapeutic effect in relation to the applied dosage can be challenging. Furthermore, TSCPC entails the risk of complications such as pain, conjunctival burn, scleral thinning, and uveitis [18,21,22,23]. Rare, but more serious, complications include hypotension, choroidal detachment, choroiditis, retinal detachment, or extremely rarely—phthisis bulbi [4]. Endoscopic cyclodestruction (ECP) is better in terms of safety and selectivity than TSCPC [24,25]. Nonetheless, it is an invasive procedure typically intended for patients with moderate glaucoma who are concurrently undergoing cataract surgery [26,27]. It is important to note that this procedure is not without side effects, with the most commonly observed ones including IOP spikes, increased inflammation (compared to phacoemulsification without ECP), and potential intraocular lens dislocation [28,29].

To improve the safety profile of cyclophotocoagulation methods developed so far, in µCPC, each laser pulse is segmented to an extremely short-duration phase. This can limit the heat accumulation and avoid coagulation temperatures, resulting in less disruption of the non-pigmented epithelium and adjacent tissues [2,4,5,6,7]. It has been proven that, compared with TSCPC, coagulative tissue damage to the ciliary body is definitely less [6,7]. However, there are no published studies describing the effect of µCPC at the intracellular level. There have been also other lowering IOP mechanisms suggested in µCPC. One considers increased uveoscleral outflow. It was directly observed in TSCPC [30,31]. In µCPC, no studies have so far shown this phenomenon; however, one study presented a variation of choroidal thickness and hypothesized that it may be indirectly caused by this effect [32]. The second proposed mechanism concerns ciliary body rotation and the subsequent opening of the angle, causing increased aqueous outflow [33]. This pilocarpine-like effect was recently proposed by Johnston et al. after an experimental study on monkeys.

Due to the fact that this is a novel procedure, the ideal laser parameters in µCPC are not decisively defined. Studies conducted so far have presented data from patients treated with energy levels ranging from 60 to 225 J. Energy (J) delivered to the eye is determined based on laser power (W), exposure duration (s), and duty cycle (%/100). Duty cycle is usually set at 31.3% for glaucoma treatment. The other two parameters are much more variable and are set based on surgeon’s preference. They vary from 1.5 W to 2.5 W and from 100 s to 360 s exposure time [11]. For a good balance between efficacy and safety, Sanchez et al. analyzed several clinical studies data and proposed mid-range level between 112 and 150 J. Using this energy level, it is possible to obtain an IOP decrease of around 35% for up to 15 months with few or no complications [10]. Lower energy level was associated with a shorter survival rate [34]. On the other hand, higher energy levels caused severe complications in more than 45% of patients and referred to persistent hypotonia, postoperative inflammation, and loss of visual acuity [13,14]. Previous studies describe a success rate of 60–80% [4,5,14,15,35,36,37,38,39,40], IOP reduction of 30–45% [4,5,15,35,36,38,40], and durability of up to 72 months with three retreatments [41]. Despite the presented earlier advantages of µCPC, efficacy and safety have to be compared to TSCPC. Only two studies have presented such results. Both authors agreed that efficacy is equal in both methods; however, µCPC has a better safety profile [5,38]. It is extremely important to emphasize that all studies on micropulse cyclophotocoagulation cited in this paper so far relate to use of Cyclo G6 810 laser with MicroPulse probe by Iridex. Laser parameters recommended by the abovementioned producer are power of 2.0–2.5 W, duty cycle of 31.3%, and from 30 to 100 s treatment time per hemisphere. 

In this study, we used a different device—diode laser FOX 810—with parameters recommended by the producer described in the method section. So far, there has only been one study published in a non-English journal where µCPC was performed on African patients with this device [8]. Authors obtained a success rate of 65% at 9 months follow-up with at least 20% IOP reduction in 20 patients with a mean energy of the device set at 127 J ± 10 J. Seven eyes did not meet the success criteria; six eyes had a further increase in IOP and one eye showed intraocular hypotension. 

This is the largest and longest study presenting the efficacy and safety of µCPC using diode laser FOX 810. In our study, a decrease in IOP and the number of antiglaucoma medications was statistically significant at each follow-up time point. The mean IOP at 18-months follow-up was reduced by 32.5%, similar to studies published so far [4,5,15,35,36,38,40]. However, due to study design, to assess the efficacy of this method, it is more valuable to analyze the number of re-interventions and success criteria. The percentage of reinterventions in the study group was 37.5% during a medium–long follow-up period. The percentage of patients who reached qualified success was moderate, at 39.2%. Compared to previously cited studies, where the success rates were 60–80%, the qualified success achieved in our study was definitely much lower, despite a similar amount of energy used. We assume that it can be explained by the lower efficacy of the device used in that study, where all published similar studies conducted considered Cyclo G6 810 laser. However, to confirm that thesis, a study should be conducted directly comparing both devices. 

The producer of the device used in our study recommends the following laser settings—power of 2 W, 31.3% duty cycle (“on” period of 500 µs/“off” period of 1.0 ms) and 90–120 s per hemisphere treatment time. The range of duration time results in total energy delivered to the eye, and differs from 111.6 J to 148.8 J considering from 90 s to 120 s per hemisphere, respectively. In our study, surgeons arbitrarily chose treatment time ranging from 180 s to 240 s based on preoperative IOP and a preoperative number of antiglaucoma medications. To be more precise in assessing the efficacy of µCPC and to determine optimal laser parameters, in the post hoc analysis we divided the study group according to the criterion of total energy used. The aim was to evaluate if energy, even in the range recommended by the producer, influenced the efficacy of the procedure. Amongst patients, where the energy used was <130 J (Group 1), the IOP at 18-month follow-up visit was reduced by 15.9%, and the percentage of reinterventions was 43.5% with a qualified success rate 31.3%. In the second group, where energy was >130 J (Group 2), IOP at 18-month follow-up visit was reduced by 38.4%, percentage of reinterventions was 36.6%, and qualified success was 42.9%. Preoperative IOP in both groups was equal (Group 1—25.1 ± 8.9 mmHg and Group 2—25.1 ± 8.3 mmHg); however, a preoperative number of antiglaucoma medications was higher in Group 2 than in Group 1 (4.4 ± 1.1 and 3.9 ± 0.6, respectively). It needs to be highlighted that, the percent of reduced IOP at the last follow-up visit in Group 1 is artificially low due to the number of patients at that visit, which is strictly related to the number of reinventions required in that group. All complications mentioned in the results section—hypotony and uveitis—happened in Group 2. Comparing results of both groups, it is noticeable that higher energy results in better efficacy and, in our opinion, 120 s treatment time per hemisphere should be used in µCPC with this device.

Upon analyzing the scatter plot of IOP (Figure 3), a noteworthy observation is that the efficacy of µCPC in lowering IOP may be influenced by the preoperative IOP level. Specifically, it was observed that preoperative IOP values below 21 mmHg did not yield a 20% reduction in IOP. This is valuable information for patient selection when considering this procedure. It is known that achieving IOP reduction becomes increasingly challenging as the preoperative IOP decreases. Additionally, among a subgroup of nineteen patients, a reduction in the number of antiglaucoma medications was observed, with the most prominent effect observed in patients with lower preoperative IOP levels. It is important to acknowledge that there are limitations in evaluating IOP, including the occurrence of regression to the mean, which is inherent to nonrandomized studies like this one. To mitigate the variability of IOP measurements, multiple assessments were conducted following the World Glaucoma Association recommendations [9]. Although this method helps reduce the impact of regression to the mean, it is still a factor that should be considered in the interpretation of the results. No significant difference was noted in visual acuity during the follow-up period. In terms of glaucoma subtypes, surgically naive patients, or patients who had undergone previous surgical interventions, we did not observed any significant differences in outcome after µCPC. 

**Figure 5:**
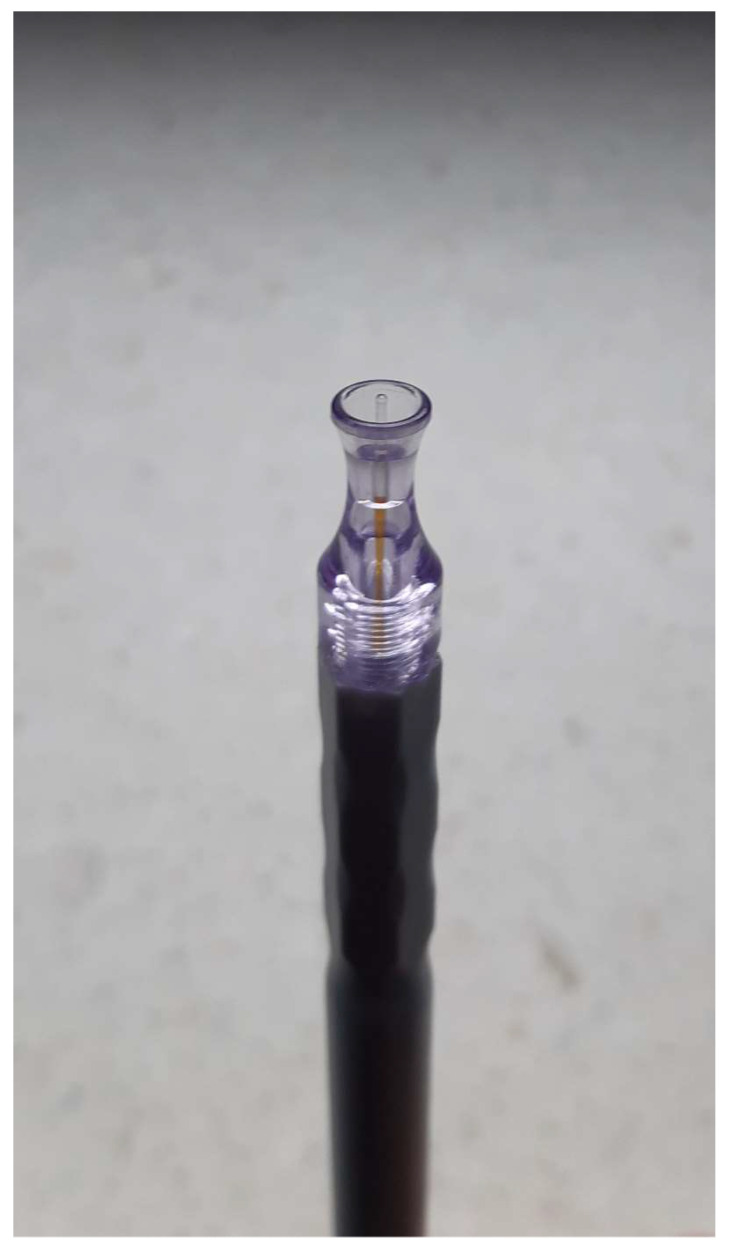
Diode laser FOX 810 probe tip (A.R.C. Laser, Nuremberg, Germany).

The most frequent complications reported were uveitis until 3 months postoperatively, loss of visual acuity, conjunctival hyperemia, hypotony, and cystoid macular edema [12]. In our study, hypotony was observed in one patient during the whole follow-up period. In this case, the intraocular pressure (IOP) was around 6 mmHg; however, the patient did not present any other ophthalmic abnormalities besides low IOP. Uveitis occurred in two patients around one month after the procedure and resolved after therapy with the topical steroid dexamethasone. Intraoperatively subconjunctival hemorrhage occurred in almost 60% of our patients, and postoperative conjunctival hyperemia in almost 70% of our patients (Figure 4). This is understandable due to the construction of the probe. The probe tip is not completely smooth and the procedure involves the probe making sweeping motions on the conjunctiva surface (Figure 5). This caused the conjunctival hyperemia and subconjunctival hemorrhage despite the use of methylcellulose. The abovementioned findings were resolved in a few weeks without any further complications. No other major intraoperative or postoperative complications occurred. There was no association between the rate of complications and whether the patients were surgically naive or non-naive. The rate of occurrence of major complications in our study was similar to other results published to date [10,11,12,13,14,15].

In summary, the mean intraocular pressure (IOP) was reduced by 32.5%; however, the success rate was moderate, likely attributable to the lower efficacy of the device used in this study compared to the one commonly utilized in the majority of micropulse TSCPC studies published thus far. No significant complications were observed following the µCPC procedure, with the exception of a few cases of uveitis and a single occurrence of hypotony. Based on our observations, despite the fact that the reintervention rate for this procedure is not low, the safety profile is high. Therefore, patients can be treated with this procedure when the target IOP reduction is not in the single-digit range and the glaucoma status allows for possible reintervention. We recommend strict follow-up, and in the case of non-responding patients, prompt reintervention without delay. Further randomized studies with a larger group of patients, longer follow-up periods, and comparison to other devices are needed to confirm these results.

## 5. Conclusions

The µCPC is well-tolerated and safe for reducing IOP in glaucoma patients in medium-term follow-up; however, success is moderate. Randomized, larger studies are needed to confirm obtained results. 

## Figures and Tables

**Table 1:** Demographic characteristics.

Age (Years), Mean (SD) [Range]	69.00 ± 14.22 [30–92]
Gender (male/female)	29/35
Type of glaucoma:	
Primary open-angle glaucoma	37
Secondary open-angle glaucoma	
Post-penetrating keratoplasty glaucoma	13
Exfoliative	0
Pigmentary	0
Uveitic glaucoma	2
Neovascular glaucoma	3
Other	2
Primary angle-closure glaucoma	4
Secondary angle-closure glaucoma	1
Aniridic glaucoma	2
Prior glaucoma surgeries:	
Trabeculectomy	9
Deep sclerectomy	1
Surgery (tube/stents)	2
Transscleral cyclophotocoagulation	16
Ultrasound Cyclo Plasty	5
Lens status:	
Phakic	35
Pseudophakic	29
Aphakic	0

**Table 2:** Intraocular pressure and number of hypotensive medications after µCPC.

Mean IOP ± SD				*p*-Value	Number of Hypotensive Medications ± SD			*p*-Value	% IOP Reduction	No. Patietns
Preop	25.1	±	8.4		4.2	±	1		-	64
1 day	17.3	±	4.5	*p* < 0.001	2.4	±	1.1	*p* < 0.001	31.2	60
1 week	16.5	±	6.1	*p* < 0.001	2.6	±	1.1	*p* < 0.001	34.4	59
1 month	20.5	±	8.3	*p* < 0.001	2.7	±	1.1	*p* < 0.001	18.6	55
3 months	17.1	±	6.2	*p* < 0.001	3	±	1.2	*p* < 0.001	32.1	46
6 months	18	±	7.1	*p* < 0.001	3	±	1.1	*p* < 0.001	28.4	35
12 months	15.8	±	3.2	*p* < 0.001	3.3	±	1	*p* < 0.001	37	30
18 months	17	±	5.9	*p* < 0.001	3.3	±	1.1	*p* < 0.001	32.5	27

**Table 3:** Intraoperative and postoperative complications after µCPC.

Complications	
**Intraoperative:**	
Pain	15/64 (23.4%)
Corneal thermal injury	0/64 (0%)
Subconjunctival hemorrhage	38/64 (59.4%)
**Postoperative:**	
Conjunctival hyperemia	44/64 (68.8%)
Epithelial defects	0/64 (0%)
Corneal edema	0/64 (0%)
Subconjunctival hemorrhage	0/64 (0%)
Hypotony, choroid detachment	1/64 (1.6%)
Retinal detachment	0/64 (0%)
Cataract	0/64 (0%)
Phthisis bulbi	0/64 (0%)
Scleral mark	0/64 (0%)
Uveitis	2/64 (3.1%)

## Data Availability

The datasets generated during and/or analyzed during the current study are available from the corresponding author on reasonable request.

## Abbreviations

IOP	Intraocular Pressure
MD	Mean Deviation
SS-OCT	Swept-Source Optical Coherence Tomography
TSCPC	Transscleral Cyclophotocoagulation
µCPC	Transscleral Microcyclophotocoagulation
UCP	Ultrasound Ciliary Plasty
